# High dose cholecalciferol supplementation causing morning blood pressure reduction in patients with type 1 diabetes mellitus and cardiovascular autonomic neuropathy

**DOI:** 10.1038/s41598-024-56934-1

**Published:** 2024-03-16

**Authors:** João Felício, Lorena Moraes, Gabriela Lemos, Ícaro Souza, Giovana Vieira, Lilian Silva, Natércia Queiroz, Ana Carolina Souza, Franciane Melo, João Felício Abrahão Neto, Hana Britto, Manuela Lemos, Márcia Santos, Priscila Figueiredo, Ana Regina Motta, Melissa Reis, Gisele Caldeira, Valéria Leal, Pedro Piani, Vitória Aquino, Karem Felício

**Affiliations:** Endocrinology Division, University Hospital João de Barros Barreto, Federal University of Pará, 4487, Guamá, Belém, Pará 66073-000 Brazil

**Keywords:** Clinical trial design, Diabetes complications, Type 1 diabetes, Neurological disorders, Cardiovascular diseases

## Abstract

We evaluated the association of cardiovascular autonomic neuropathy (CAN), blood pressure (BP) and Vitamin D (VD) levels before and after high-dose cholecalciferol supplementation (4000/10,000) UI/day) for 12 weeks in patients (N = 67) with type 1 diabetes mellitus (T1DM). Based on this prospective controlled pilot study, patients were divided into group 1 (N = 23 with CAN) and group 2 (N = 44 without CAN). At baseline, group 1 had higher systolic BP (SBP) during sleep (115 ± 14 vs. 107 ± 12 mmHg, *p* = 0.04) and lower nocturnal dipping (3 ± 5 vs. 8 ± 6%, *p* = 0.009). Among those with loss of nocturnal dipping, 45.4% (20/44) had CAN, while in normal nocturnal dipping group it occurred only in 13% (3/23) (*p* = 0.007). Non-dipper group had worse CAN parameters when compared to dipper group [Very low frequency (VLF) (2.5 ± 0.5vs.2.8 ± 0.4 s, p = 0.01), total power (TP) (2.9 ± 0.6 vs. 3.3 ± 0.4 s, *p* = 0.01), Valsalva coefficient (1.5 ± 0.4 vs. 1.8 ± 0.6, *p* = 0.06)]. After VD, only group 1 improved CAN parameters [TP (2.5 ± 0.4 vs. 2.8 ± 0.6, *p* = 0.01) and VLF (2.2 ± 0.4 vs. 2.4 ± 0.5, *p* = 0.03). Group 1 presented a reduction in morning SBP (120 ± 20 vs. 114 ± 17 mmHg, *p* = 0.038) and in morning SBP surge (13 ± 13 vs. 5 ± 14, *p* = 0.04). High-dose VD was associated with improved CAN parameters and reduced awake SBP and morning SBP surge. These findings suggest that VD may benefit patients with cardiovascular autonomic neuropathy. ISRCTN32601947, registration date: 31/07/2017.

## Introduction

Diabetic neuropathy is a serious and common complication of diabetes mellitus (DM), the prevalence reports vary from 10 to 90% in DM patients, depending on the diagnostic methodology used. Clinical manifestations relate to the type of nerve fiber affected^1,2^. Cardiovascular autonomic neuropathy (CAN) is a clinically important form of diabetic autonomic neuropathy (DAN) and was defined in the Toronto Consensus as the impairment of autonomic control of the cardiovascular system in patients with established DM following the exclusion of other causes^3^. CAN is associated with mortality independently of other cardiovascular risk factors and has a variable clinical presentation^4,5^. In its early stages, CAN may be asymptomatic and detected only by decreased heart rate variability with deep breathing^6^. Advanced disease may be associated with resting tachycardia and orthostatic hypotension, upon standing without an appropriate increase in heart rate^6^.

Autonomic dysfunction and abnormal blood pressure (BP) variability have been associated with a bad prognosis for cardiovascular diseases^7^. Systematics reviews^8,9^ suggest associations between the presence of CAN with loss of nocturnal dipping and increases in morning BP and in morning BP surge. However, in Type 1 DM (T1DM) patients, the influence of CAN in blood pressure remains unclear, since few cross-sectional and longitudinal studies address this issue^10–12^ and there is no consensus among them^13,14^.

Furthermore, the role of vitamin D (VD) in autonomic cardiovascular balance and in blood pressure maintenance have been discussed^15–18^. Regarding this matter, Mann et al.^15^ found a suppression of sympathovagal balance in healthy and normotensive patients with 25(OH)D deficiency, while our group found a reduction in morning BP in normotensive T1DM patients after high-dose VD supplementation^16^. Additionally, in patients with diabetes, insufficient VD levels were associated with lower parasympathetic activity^17^, while VD supplementation was associated with improved CAN parameters in a previous study by our group^18^. Furthermore, a recent review suggests that VD supplementation may be used to slow or stop the progression of neural damage in DM patients^19^.

Therefore, this study aims to evaluate the association between cardiovascular autonomic neuropathy, blood pressure levels and vitamin D levels in T1DM patients before and after supplementation with high doses of cholecalciferol.

## Materials and methods

### Study design and patients

We performed a prospective controlled pilot study with 67 T1DM patients for 12 weeks, before and after cholecalciferol supplementation (4000/10,000 UI/day) to evaluate a possible benefit in morning blood pressure with or not associated to cardiovascular autonomic neuropathy, as part of a research protocol registered on 31/07/2017 (ISRCTN32601947) that has already provided evidence on other effects of VD supplementation in T1DM^16,18,20–22^.

The study was developed according to the Declaration of Helsinki and Nuremberg Code and was approved by the University Hospital João de Barros Barreto research ethics committee, reference number 0122.0.071.000-12, in accordance with the standards of the National Health Council. Written and informed consent was collected from all patients included in this study.

This trial is an extension of Silva et al.^18^ which evaluated the effect of high-dose vitamin D (VD) supplementation on CAN in Type 1 Diabetes Mellitus (T1DM) patients. In addition to the 23 T1DM CAN patients already analyzed in the previous study, 44 T1DM patients without CAN were recruited to form a control group, totalizing 67 subjects, from both sexes and different age groups, recruited from the endocrinology ambulatory and enrolled in this study.

VD supplementation dosage was decided according to basal VD levels, with the aim of maintaining serum levels above 30 ng/mL and below 100 ng/mL^23^. Individuals with 25(OH)D levels between 30 and 60 ng/mL have received 4000 IU/day of cholecalciferol and those with deficiency and/or insufficiency (< 30 ng/mL) have received 10,000 IU/day for 12 weeks.

At the beginning of the study, patients were divided into two groups according to the presence (N = 23) and the absence of CAN (N = 44). Subsequently, the total patients were redivided, in a post hoc analysis, into Dipper (nocturnal dipping > 10%) and Non-dipper (nocturnal dipping < 10%) groups, for better analysis the effects of CAN on the loss of nocturnal dipping.

Main inclusion criteria consisted in: (a) patients with T1DM diagnosis in at least a 1-year follow-up; (b) age between 12 and 50 years in regular treatment with an endocrinologist; (c) glycated hemoglobin (HbA1C) ≥ 7%; (d) insulin therapy dose stability at least 3 months before participating in the study; (e) NPH, Glargine, Detemir, Aspart, Glulisin, Lispro, and Regular insulin were insulins allowed; (f) patient in use of metformin could participate of the study as long as they were using the same dose for at least 3 months; (g) compliance with diet and exercise regimen; (h) ACEI or ARB (for diabetic kidney disease) doses must show stability for at least 3 months before participating in the study. Patients were instructed to maintain diet and physical activity according to guidelines of American Diabetes Association^24^ to participate in this study.

Main exclusion criteria included: history of (a) hepatic diseases; (b) bone metabolism disorders and previous VD or Calcium supplementation; (c) abnormal serum creatinine levels (d) anemias; (e) pregnancy or breastfeeding women; (f) uncontrolled hypo or hyperthyroidism and allergies to VD supplementation elements.

### Data collection

Data collection occurred during schedule visits at baseline, during and at the end of study. There were 4 official visits in the trial and extra visits if necessary. Patients were recruited from the endocrinology ambulatory division and, in visit 1, after the evaluation of inclusion and exclusion criteria and signed written informed consent, medical records (pre-existent clinical conditions, demographics, insulin and other medications in use), physical examination, laboratorial, CAN tests and ABMP were performed. Clinical aspects, laboratory analysis, ambulatory blood pressure monitoring (ABPM) and tests for CAN evaluation were executed before and after 12 weeks. Medical records (pre-existent clinical conditions, demographics, insulin and other medications in use) and physical examination were performed.

Dyslipidemia was defined according to the Canadian Cardiovascular Society guidelines^25^. Hypertension was defined according to American Heart Association guideline^26^. Retinopathy, nephropathy, and peripheral neuropathy were evaluated accosting to American Diabetes Association guidelines^27,28^.

Serum 25(OH)D was measured by DiaSorin LIAISON 25-OH-Vitamin D TOTAL chemiluminescence immunoassay (DiaSorin, Stillwater, MN, USA)^29^. DiaSorin LIAISON is one of the methods to evaluate 25(OH)D tested by DEQAS (Vitamin D External Quality Assessment Scheme), the largest specialist external quality assessment (proficiency testing) scheme for the vitamin D metabolites 25(OH)D and a 1.25(OH) 2D. The method has 100% recovery of vitamin D2 and vitamin D3 which allow the optimal characterization of the nutritional status and of the 25(OH)Vitamin D supplementation^30^.

HbA1c was measured by high-performance liquid chromatography (HPLC)^31^. Fasting glucose, plasma glucose, urea, albumin, total cholesterol, low-density lipoprotein cholesterol (LDL-C), high-density lipoprotein cholesterol (HDL-C), and triglycerides were measured by colorimetry. Ultrasensitive C-reactive protein (PCR-US) was measured by nephelometry. Creatinine was measured by colorimetric method. Glomerular filtration rate (GFR) was calculated by Chronic Kidney Disease Epidemiology Collaboration equation (CKD-EPI)^32^.

### CAN evaluation

Parameters used to diagnose and evaluate CAN were Very Low Frequency (VLF), Low Frequency (LF), High Frequency (HF), respiratory coefficient, 30/15 coefficient and Valsalva coefficient, as well as systolic blood pressure (SBP) reduction in orthostasis. These tests have high sensitivity and specificity (97.3 and 96.2%, respectively), and low coefficients of variation, such as 9.2, 12.6 and 6.4% for the Valsalva maneuver, deep breathing test and lying-standing test, respectively^33^. According to the Toronto statement, the presence of one abnormal parameter is defined as possible CAN or early CAN; two abnormal parameters is defined as confirmed CAN, and the presence of orthostatic hypotension is defined as severe CAN^3^. Clinical conditions as alcohol abuse, Hansen’s disease, vitamin B12 deficiency and hypoglycemia were excluded as causes of CAN. All procedures were performed before and after vitamin D supplementation. The tests were always made in the morning. Participants were instructed not to perform vigorous physical exercises 24 h before examination and not to use alcohol, caffeine beverages and tobacco for at least 8 h before the test. Fasting capillary glycemia levels were maintained between 70 and 250 mg/dl.

Seven heart rate variability (HRV) parameters (three in rest and four while performing stimulatory maneuvers)^34^ were analyzed by VNS-MICRO software (Neurosoft, Ivanovo, Russia). With the patient in supine position, an electrocardiographic record was performed for 300 s. R waves are highlighted by the software and each regular RR interval is analyzed by an algorithm and then expressed through an amplitude diagram of HR oscillation (HR fluctuations per second) versus HR in hertz. Total amplitude of HRV spectrum was distributed in three bands: VLF component (0.01–0.04 Hz), which is related to vasomotor tonus fluctuations linked to thermoregulation and sweating (sympathetic control); LF component (0.4–0.15 Hz), associated with baroreceptor reflex; and HF component (0.15–0.5 Hz), related to parasympathetic control (vagus nerve). The software also provided other data about rest HRV, such as: RRmin (minimum RR interval), RRmax (maximum RR interval), RRNN (mean length of regular RR intervals), and SDNN (standard deviation of all NN intervals)^35,36^.

Frequency domain parameters were composed of: VLF, HF, LF and Total Power (TP), a set of three combined spectral bands and LF/HF ratio (which reflects balance between sympathetic and parasympathetic systems). Although it is not a diagnostic criterion, it provides additional information on sympathetic and parasympathetic performance in heartbeat. Time domain parameters were composed of: RRmin, RRmax, RRNN and SDNN.

The stimulatory maneuvers used were deep breathing, Valsalva and orthostasis (blood pressure and HRV). In each test the relation between the largest and smallest RR interval is assessed and then, a coefficient was obtained.

### ABPM evaluation

The oscillometric method was used to perform non-invasive 24 h-ABPM, placed in the morning and carried over for 24 h. Participants were instructed to maintain their usual occupations and to note the time and the description of each daily activity. Systolic and diastolic BP (SBP and DBP) means were instituted for each hour, during the waking and sleep period, as well as throughout 24 h, since the machine executed a BP check every 15 min. 24-h ABPM has good reproducibility and low coefficient of variation among patients with diabetes^37^. Morning BP and sleep-through morning BP surge was defined as the average of BP values of the first 2 h after awakening and the difference between the morning pressure and the arithmetic mean between the lowest BP and the pressures immediately before and after the lowest BP, respectively^38^. Nocturnal blood pressure dipping was calculated by the following formula: Systolic or diastolic nocturnal dipping (%) = (mean SBP/DBP in waking time − mean SBP/DBP during sleep) × 100/average systolic BP in waking time^37^. Due to technical issues, thirteen patients were not able to perform ambulatory blood pressure monitoring after vitamin D supplementation.

### Statistical analysis

Categorical variables were described as frequency (percentage). Numerical variables with normal distribution were described as mean and standard deviation, while variables with non-normal distribution were described as median and interquartile range. Normality was stablished using Shapiro–Wilk. T-Student and Man-Whitney tests were used to compare two groups with numerical variables with and without normal distribution, respectively. Wilcoxon and paired T-test were used in the comparison between variables of the same groups before and after the follow-up period, and chi-square for binomial variables. If we needed to compare more than two variables before and after, ANOVA of repeated measures or Friedman test were used. For correlation analysis, Pearsons and Spearman's tests were used. Frequency domain parameters were expressed in logarithm with base 10. Sensitivity was calculated by the formula: [a/(a + c)] × 100; specificity was calculated by the formula [d/(b + d)] × 100. In which “a” means true positive, “b” false positive, “c” false negative and “d” true negative.

Our sample size was calculated based on an expected power of 0.8 to detect the difference before and after between the parameters of CAN (HF, VLF, LF and Valsalva coefficient) and the number of patients necessary was 20 cases with baseline cardiovascular autonomic neuropathy. Since this subgroup was composed by 23 patients, the power of our study to detected improvements in CAN parameters was > 0.8 and considered satisfactory.

Data collected were organized and analyzed by SigmaPlot 12.0 (Systat Software, Chicago, IL) and SPSS Statistics 22® (IBM Corp., Armonk, NY, USA) programs. *P* values < 0.05 were considered significant.

## Results

Our sample was composed of 67 patients with type 1 diabetes mellitus, of both sexes, who received vitamin D supplementation. At baseline, 21/67 (31%) patients had abnormal albuminuria (16 with moderately increased and 5 with severely increased albuminuria). Additionally, only 11 patients had non-proliferative retinopathy. In the group of patients with cardiovascular autonomic neuropathy, 9 had lower to moderate alcohol consumption vs 13 patients in the group without cardiovascular autonomic neuropathy (*p* = 0.6; NS). The clinical and laboratory characteristics are shown in Table 1.

**Table 1:** Clinical characteristics of patients with Type 1 Diabetes Mellitus.

Characteristics	N = 67
Age (years)	28 ± 10
Sex (Female/Male)	34/33
T1DM duration (years)	12 ± 8
Dyslipidemia (yes/no, %)	18/49 (27%)
Systemic Arterial Hypertension (yes/no, %)	11/56 (16.4%)
Nephropathy (yes/no, %)	21/46 (31.3%)
Retinopathy (yes/no, %)	11/56 (16%)
Peripheral neuropathy (yes/no, %)	17/50 (25%)
Smoking (yes/no, %)	11/56 (16%)
Alcoholism (yes/no, %)	22/45 (32.8%)
ACEI/ARB previous use (yes/no, %)	20/47 (30%)

*T1DM* type 1 diabetes mellitus, *ACEI* angiotensin-converting enzyme inhibitor, *ARB* angiotensin receptor blocker.

To better evaluate the influence of cardiovascular autonomic neuropathy (CAN) on blood pressure, the patients were divided into two groups according to the presence (N = 23) or absence (N = 44, control group) of CAN. In both groups ABPM was performed before and after VD supplementation. At baseline, CAN patients had higher systolic sleep blood pressure (115 ± 14 vs. 107 ± 12 mmHg, *p* = 0.04) and lower nocturnal dipping (SBP 3 ± 5 vs. 8 ± 6 mmHg, *p* = 0.009; DBP 6 ± 7 vs. 11 ± 8 mmHg, *p* = 0.019) when compared to the group without CAN.

**Figure 1:**
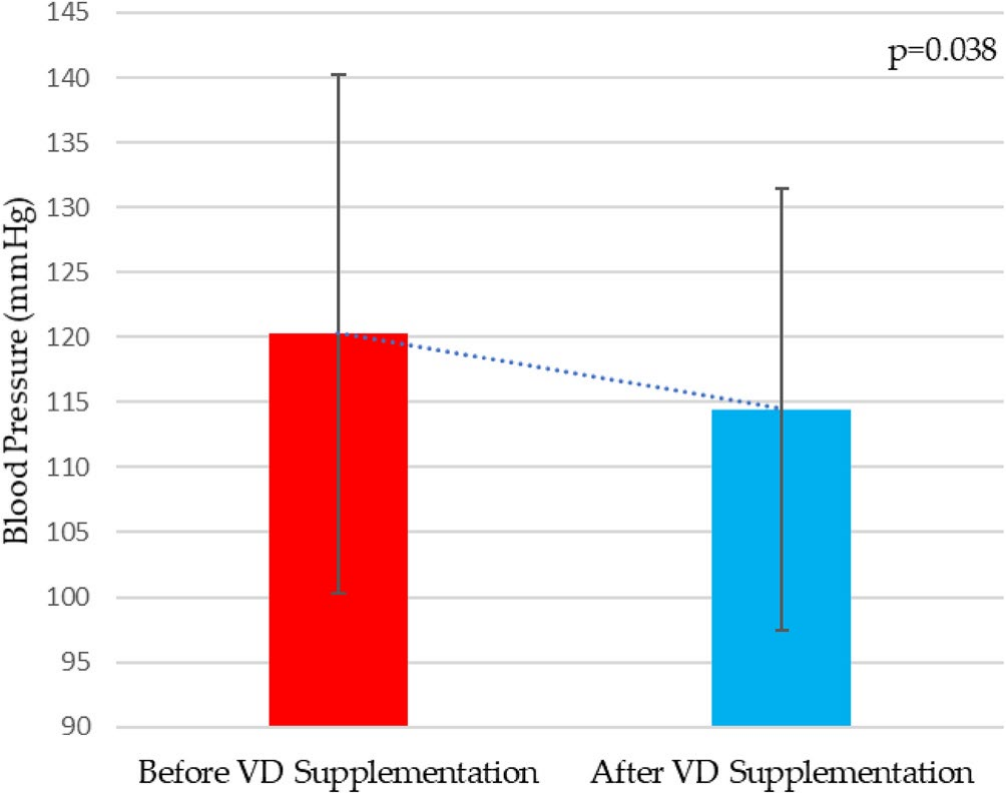
Morning systolic blood pressure before and after high-dose vitamin D supplementation in patients with CAN. VD, vitamin D.

**Figure 2:**
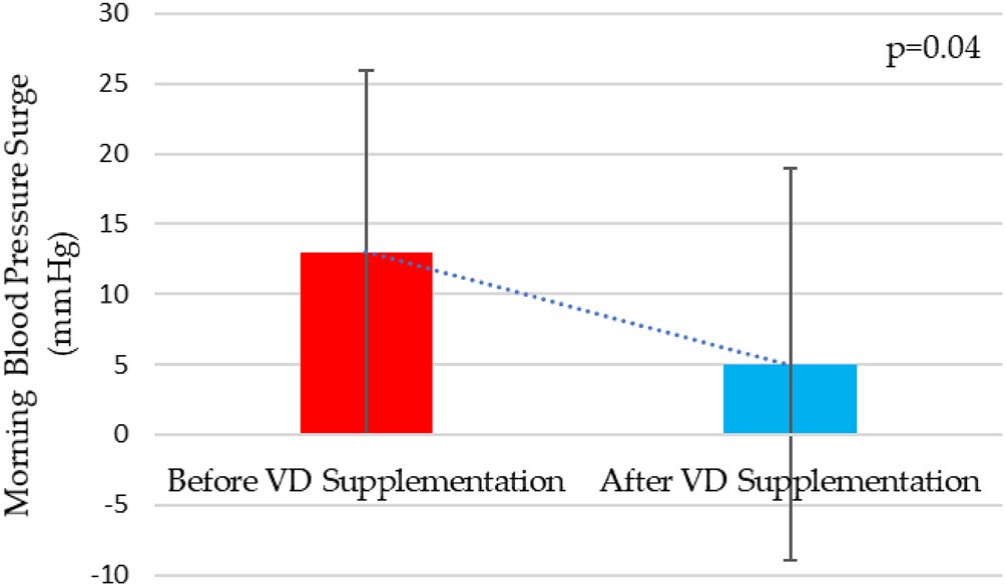
Morning blood pressure surge before and after high-dose vitamin D supplementation in patients with CAN. VD, vitamin D.

After Vitamin D supplementation, CAN group had a significant reduction of total (118 ± 13, vs. 115 ± 12 mmHg, *p* = 0.006) awake (119 ± 12 vs. 115 ± 13 mmHg, *p* < 0.001) and morning systolic blood pressure (120 ± 20 vs. 114 ± 17 mmHg, *p* = 0.038) (Fig. 1), including a drop in morning blood pressure surge (13 ± 13 vs. 5 ± 14 mmHg, *p* = 0.04) (Fig. 2). Besides that, no improvement was observed in systolic nocturnal dipping in these patients. Furthermore, in the group without CAN, there were no changes in the parameters analyzed by ABPM after VD supplementation.

At baseline, 25(OH)D levels were negatively correlated with the presence of cardiovascular autonomic neuropathy (r = − 0.254, *p* = 0.04). Additionally, CAN parameters had a positive correlation with nocturnal dipping (TP: r = 0.4 *p* = 0.002; VLF: r = 0.4 *p* = 0.003; HF: r = 0.4 *p* = 0.003).

No changes were found in parameters of glycated hemoglobin, fasting glucose, lipid profile or other clinical and laboratory aspects after VD supplementation. As expected, 25(OH)D levels were elevated (Table 2). In the beginning of the study, 16/67 (23.8%) patients had VD deficiency (levels < 20 ng/mL). After 12 weeks of cholecalciferol supplementation, only 2/67 (2.9%) remained with the deficiency.

**Table 2:** T1DM patients clinical and laboratorial characteristics before and after vitamin D supplementation.

Characteristics	N = 67		*p*
	Pre VD Mean ± SD	Post VD Mean ± SD	
BMI (kg/m^2^)	24 ± 4	24 ± 4	NS (0.94)
HbA1C (%)	9.5 ± 2.3	9.7 ± 2.6	NS (0.92)
Basal insulin (UI)	35 ± 17	36 ± 17	NS (0.93)
Prandial insulin (UI)	22 ± 11	23 ± 11	NS (0.60)
25-OH-vitamin D (ng/mL)	26 ± 9	53 ± 24	< 0.001
Fasting glucose (mg/dL)	165 ± 94	179 ± 101	NS (0.41)
Ultra-sensitive CRP (mg/dL)	0.35 ± 0.5	0.36 ± 0.53	NS (0.80)
Total cholesterol (mg/dL)	171 ± 40	180 ± 59	NS (0.99)
HDL cholesterol (mg/dL)	51 ± 37	44 ± 10	NS (0.26)
LDL cholesterol (mg/dL)	103 ± 30	108 ± 48	NS (0.84)
Triglycerides (mg/dL)	99 ± 56	108 ± 70	NS (0.65)
Creatinine (mg/dL)	0.8 ± 0.2	0.8 ± 0.2	NS (0.61)
Heart rate (bpm)	83 ± 13	82 ± 14	NS (0.73)

*VD* vitamin D, *BMI* body mass index, *HbA1C* glycated hemoglobin, *HDL* high density lipoprotein, *LDL* low density lipoprotein, *NS* non-significant, *SD* standard deviation.

CAN parameters performed are described in Table 3. As described in previous works^18^, an improvement was observed in the frequency domains (VLF, HF, LF and TP) and in RRmax, RRNN and SDNN in the group with CAN.

**Table 3:** CAN parameters before and after vitamin D supplementation in groups with and without CAN.

Parameter	CAN (N = 23) Mean ± SD		Without CAN (N = 44) Mean ± SD		*p*
	Pre VD	Post VD	Pre VD	Post VD	
Frequency domain parameters					
VLF (log10 sec)	2.2 ± 0.4	2.4 ± 0.5	2.94 ± 0.37	2.8 ± 0.36	0.03*
LF (log10 sec)	1.9 ± 0.5	2.5 ± 0.9	2.8 ± 0.4	2.8 ± 0.4	< 0.001*
HF (log10 sec)	1.7 ± 0.5	2.2 ± 0.8	2.8 ± 0.4	2.8 ± 0.57	0.01*
TP (log10 sec)	2.5 ± 0.4	2.8 ± 0.6	3.39 ± 0.38	3.3 ± 0.4	0.01*
Cardiac autonomic reactivity tests					
Respiratory coefficient	1.2 ± 0.3	1.2 ± 0.2	1.44 ± 0.24	1.36 ± 0.2	0.007^
Valsalva coefficient	1.4 ± 0.4	1.5 ± 0.6	1.77 ± 0.5	1.6 ± 0.4	NS (0.8*; 0.1^)
30/15 coefficient	1.2 ± 0.3	1.2 ± 0.2	1.38 ± 0.2	1.5 ± 0.9	NS (0.3*; 0.6^)
3 min SBP reduction	6.9 ± 14.1	9.2 ± 14.6	− 0.7 ± 6.4	− 0.7 ± 7	NS (0.6*; 0.8^)
Time domain parameters					
RRmin (sec)	0.66 ± 0.094	0.62 ± 0.16	0.66 ± 0.19	0.58 ± 0.19	0.02^
RRmax (sec)	0.77 ± 0.11	0.94 ± 0.51	1.07 ± 0.3	1.0 ± 0.24	0.008*
RRNN (sec)	0.71 ± 0.1	0.76 ± 0.09	0.9 ± 0.2	0.8 ± 0.14	0.01*; 0.02^
SDNN (sec)	0.02 ± 0.01	0.03 ± 0.02	0.05 ± 0.02	0.04 ± 0.02	0.002*

*Before x after, CAN group; ^Before x after, without CAN group.

*CAN* cardiovascular autonomic neuropathy, *SD* standard deviation, *VLF* very low frequency, *LF* low frequency, *HF* high frequency, *TP* total power, *SBP* systolic blood pressure, *RRmin* minimum RR interval, *RRmax* maximum RR interval, *RRNN* mean length of regular RR intervals, *SDNN* standard deviation of all NN intervals, *NS* non-significant.

Source: adapted from Silva et al. ^18^.

In addition, patients were divided into dipper and non-dipper groups to analyze CAN effects on the loss of nocturnal dipping. Within the non-dipper group, the CAN prevalence was 45.4% (20/44), while in the dipper group it was 13% (3/23) (*p* = 0.007). At baseline, the dipper group had better CAN parameters values when compared to non-dippers (Table 4).

**Table 4:** Comparison between basal CAN parameter values between Dipper and Non-Dipper groups.

Parameter	Dipper (23) Mean ± SD	Non-dipper (44) Mean ± SD	*p*
Frequency domain parameters			
VLF (log10 sec)	2.8 ± 0.4	2.5 ± 0.5	0.01
LF (log10 sec)	2.7 ± 0.4	2.3 ± 0.6	0.04
HF (log10 sec)	2.8 ± 0.5	2.2 ± 0.7	0.006
TP (log10 sec)	3.3 ± 0.4	2.9 ± 0.6	0.01
Cardiac autonomic reactivity tests			
Respiratory coefficient	1.5 ± 0.3	1.2 ± 0.2	0.01
Valsalva coefficient	1.8 ± 0.6	1.5 ± 0.4	0.06
30/15 coefficient	1.3 ± 0.2	1.3 ± 0.3	NS (0.5)
3 min SBP reduction	− 0.9 ± 6	4 ± 11	NS (0.09)
Time domain parameters			
RRmin (sec)	0.68 ± 0.17	0.65 ± 0.15	NS (0.4)
RRmax (sec)	1.07 ± 0.37	0.9 ± 0.23	0.02
RRNN (sec)	0.87 ± 0.16	0.8 ± 0.16	0.02
SDNN (sec)	0.05 ± 0.03	0.03 ± 0.02	0.01

*CAN* cardiovascular autonomic neuropathy, *SD* standard deviation, *VLF* very low frequency, *LF* low frequency, *HF* high frequency, *TP* total power, *SBP* systolic blood pressure, *RRmin* minimum RR interval, *RRmax* maximum RR interval, *RRNN* mean length of regular RR intervals, *SDNN* standard deviation of all NN intervals, *NS* non-significant.

There was an improvement in CAN parameters only in the non-dipper group, causing loss of statistical difference when comparing the two groups after VD supplementation (Table 5).

**Table 5:** Comparison between CAN parameter values between Dipper and Non-Dipper groups after vitamin D supplementation.

Parameter	Dipper (20) Mean ± SD	Non-dipper (34) Mean ± SD	*p*
Frequency domain parameters			
VLF (log10 sec)	2.8 ± 0.3	2.6 ± 0.5	NS (0.055)
LF (log10 sec)	2.8 ± 0.5	2.6 ± 0.7	NS (0.14)
HF (log10 sec)	2.8 ± 0.6	2.4 ± 0.6	NS (0.17)
TP (log10 sec)	3.3 ± 0.4	3 ± 0.6	NS (0.07)
Cardiac autonomic reactivity tests			
Respiratory coefficient	1.4 ± 0.2	1.2 ± 0.18	0.036
Valsalva coefficient	1.6 ± 0.5	1.5 ± 0.5	NS (0.27)
30/15 coefficient	1.6 ± 1.2	1.3 ± 0.2	NS (0.06)
3 min SBP reduction	− 2.3 ± 6.8	2.5 ± 8.6	0.022
Time domain parameters			
RRmin (sec)	0.53 ± 0.20	0.64 ± 0.19	0.04
RRmax (sec)	0.98 ± 0.19	0.96 ± 0.29	NS (0.68)
RRNN (sec)	0.82 ± 0.13	0.80 ± 0.14	NS (0.52)
SDNN (sec)	0.05 ± 0.03	0.04 ± 0.02	NS (0.11)

*CAN* cardiovascular autonomic neuropathy, *SD* standard deviation, *VLF* very low frequency, *LF* low frequency, *HF* high frequency, *TP* total power, *SBP* systolic blood pressure, *RRmin* minimum RR interval, *RRmax* maximum RR interval, *RRNN* mean length of regular RR intervals, *SDNN* standard deviation of all NN intervals, *NS* non-significant.

When the nocturnal dipping absence (non-dipper) was used as a marker of CAN, it presented sensitivity and specificity of 90 and 45%, respectively.

After VD supplementation, positive correlations were also found between CAN frequency domains and nocturnal dipping (TP: r = 0.6 *p* < 0.001; VLF: r = 0.5 *p* < 0.001; HF: r = 0.6 *p* < 0.001). It was also found a positive correlation between SDNN and absolute 25(OH)D levels (r = 0.245, *p* = 0.04).

## Discussion

Our study found reductions in total, morning, awake and morning surge SBP, in addition to an improvement in CAN parameters in patients with TDM1 and CAN after high doses of cholecalciferol supplementation. This did not occur in our control group without CAN. Furthermore, associations were found between 25(OH)D levels, CAN, and nocturnal dipping in TDM1 patients. Finally, at baseline, the non-dipper group had higher CAN prevalence when compared to the dipper group and, after cholecalciferol supplementation, improvements in CAN parameters occurred mainly in the first group, minimizing the differences found between them at the beginning of the study.

Recently our group described that, in TDM1 and CAN patients, cholecalciferol supplementation can improve CAN indexes^18^. Simultaneously, data from our group suggests a beneficial effect of this supplementation by decreasing awake and morning blood pressure in normotensive TDM1 patients^16^. Our data, evaluating a larger group of patients, reinforce these findings by associating vitamin D levels, CAN, and loss of nocturnal dipping in 24-h blood pressure rhythm.

Although the effect of VD in CAN is multifactorial, inflammatory pathway modulation and regulation of neurotrophins can explain this relationship^39^. About the first mechanism, lower VD levels are associated with increased inflammatory markers in DM patients [C-reactive protein (CRP), expression of 2 and 4 toll-like receptors, interleukins, and κB nuclear factor (NF-kB)]^40^. We found no CRP reduction in our study. Shih et al. in agreement to our findings, studying 25 patients with T1DM also failed by demonstrating inflammatory markers decrease after VD supplementation^41^. This might have occurred due to our study duration, whether a longer time of VD sufficient levels might be necessary to affect those inflammatory markers. Another possibility is that the effect of VD on inflammatory process occurred only in presence of very low VD values. In our study, at baseline, only 16/67 (23.8%) patients had VD levels < 20 ng/ml. Anandabaskar et al.^42^, in a clinical trial, related VD supplementation with improved vascular function and reduced oxidative stress in patients with type 2 DM patients^42^. Finally, it is also known that DM patients have an increased production of reactive oxygen species (ROS), which depresses synaptic transmission of the autonomic ganglion and increases the risk of cardiac arrhythmias in DM patients with CAN^43^.

About the second possible mechanism, neurotrophins are proteins responsible for the development, maintenance and functioning of the nervous system^44^. Vitamin D insufficiency is related to neurotrophins reduction, increasing risk of toxic and metabolic nerve damage^45^. Also, VD is shown to have a neuroprotective effect involved with neurotrophins regulation, mediated by VDR on neurons and glia cells which interacts with g-aminobutyric acid (GABA) and glutamatergic neurotransmission, suppressing inflammation and oxidative stress^39,46^.

Heart rate variability (HRV) analysis is one of the most sensitive measures available for the evaluation of autonomic function and several studies pointed out that several parameters are significantly reduced in diabetic patients with CAN^7,47^. Razanskaite-Virbickiene et al.^48^, in a case control study, demonstrated the reduction of overall HRV parameters in young T1DM patients with confirmed CAN, especially in deep breath^48^. Furthermore, the coefficients of variation (time domain analysis parameter) for CAN diagnosis in deep breath had better results of sensitivity and specificity, respectively 97.3 and 96.2%. Additionally, Pop-Busui et al.^49^, in a clinical trial with TDM1 patients from the DCCT/EDIC study, found an agreement between the results of the CART tests and HRV time and frequency domains, suggesting that electrocardiogram in CAN diagnosis may be an accessible option in clinical practice. In line with these findings, at baseline, our study found reduced values in frequency and time domains in CAN patients and an association between them and loss of nocturnal dipping. Subsequently, an improvement in these values was observed in patients with CAN after VD supplementation, mainly in the non-dipper group, minimizing the differences between them and the dipper group found at baseline, which may suggest a positive effect of VD on autonomic dysfunction.

Systematic reviews^8,9^ suggest that rises in morning blood pressure may be related to autonomic nervous system dysregulation, and presence of CAN is associated with both loss of nocturnal dipping and high values of pressure and morning surge. In a cross-sectional study of 167 diabetic patients, Di Gennaro et al.^10^ found an independent association between CAN presence and high values of morning blood pressure surge. In addition, Lodhi et al.^50^, in a case–control study, observed an association between increased variability of awake BP and presence of autonomic dysfunction, specifically the awake SBP standard deviation showed greater diagnostic value than nocturnal dipping. In our study, after VD supplementation the CAN patients had a significant reduction in morning surge and morning SBP, associated with improvement in CAN parameters. Some hypothesis could justify the abrupt drop in morning SBP in our patients. Therefore, the suppression of the RAAS and cortisol arise as possible candidates^51^. The effects on SBP and morning surge through these pathways may be more immediate and may reduce cardiovascular risk in short term^51^. Furthermore, as we are aware, this is the first study to find an abrupt reduction in morning blood pressure and morning surge SBP in hypertensive and normotensive TDM1 patients with CAN after high-dose cholecalciferol supplementation.

Other factors can influence morning blood pressure levels, such as cortisol and aldosterone concentrations. Muscogiuri et al.^52^ state that there is an inverse relationship between VD and cortisol, in addition to demonstrating that VD metabolites and adrenocortical hormones share some metabolic pathways and a possible influence of polymorphisms in genes related to VDR. As for aldosterone, there are differing opinions regarding the effects of VD on RAAS. While animal studies discuss that low VD levels may be associated with increased RAAS activity resulting in cardiovascular disease and hypertension^53^, cross-sectional studies and clinical trials with VD supplementation have shown negative results regarding this influence^54,55^, showing that this association is still on debated and has not been proven yet in the literature**.** In our study, it was not possible to measure cortisol and aldosterone levels, which is a limitation of the work. Thus, further studies are necessary to address these hormones and other components of the RAAS to assess their relationship with vitamin D.

In addition to the known factors in the development and progression of CAN, such as diabetes duration and glycemic control, other aspects have been investigated^56,57^. Braffet et al.^57^, in the DCCT/EDIC study, found that higher HbA1c, heart rate and BP, β-blocker use, sustained albuminuria, GFR < 60 mL/min/1.73 m^2^ in TDM1 patients are the most significant risk factors for the development of CAN. Furthermore, Andersen et al.^56^, in the ADDITION-Denmark prospective study that followed DM2 patients for 6 and 13 years, observed obesity, hypertriglyceridemia and hyperglycemia as risk and worsening factors of CAN^56^. Overmore, they also found an improvement in CAN parameters during the follow-up period, which the authors attribute to the multifactorial treatment and early diagnosis of CAN^56^. Therefore, instruments that can contribute to early CAN diagnosis are necessary, since the disease in its initial stages presents itself in a subclinical form, and the analysis of BP variability with ABPM is an available option and recently suggested in the literature for this purpose^6,50^. Therefore, when seeking for BP changes triggered by dysautonomia, such as those found in our study, this test can help to delay the progression of the disease from an early diagnosis.

Recent studies discuss about the use of non-dipper and reverse dipper pattern of nocturnal dipping as possible markers of CAN presence, however, data in literature are contradictory^9,12–14^. In our study, we observed, in a post-hoc analysis at baseline, an association between non-dipper patients and autonomic function tests, with high prevalence of CAN patients within this group, in addition to a correlation between CAN tests and nocturnal dipping before and after VD supplementation. Chiriacò et. al.^12^, in a longitudinal retrospective study, evaluated the prognostic value of ABPM in TDM1 and DM2 patients, and found that the presence of “non-dipper” e “reverse dipping” patterns were associated with a higher prevalence of autonomic cardiovascular neuropathy, chronic kidney disease, and to an increased risk of all-cause mortality. In contrast, Jaiswal et al.^13^, in a longitudinal study, and Stella et al.^14^, in a cross-sectional study, did not find associations between the non-dipper pattern and tests for CAN diagnosis; however, in both studies, the non-dippers prevalence among TMD1 patients was lower in comparison to our group (10 vs. 65%). These findings may corroborate the possibility that nocturnal dipping may be used as a screen tool for CAN. Nevertheless, the low specificity indicates a need to an additional test to confirm the diagnosis, but it could still be useful to select asymptomatic patients with diabetes to perform reflex tests.

The main limitation of our study are the absence of randomization and placebo group which are necessary to stablish definitively if high-dose vitamin D supplementation could an effect agent of intervention. Our study has a small number of patients with T1DM and CAN, but the power of the tests to detect differences in CAN parameters was adequate. We did not measure cortisol and aldosterone levels which could clarify possible VD mechanisms of action. Finally, we used immunoassay to measure vitamin D instead of mass spectrometry. which is the gold standard. Since this is a pilot study, prospective and large-scale clinical trials are necessary to clarify our findings.

## Conclusions

Our data showed that vitamin D supplementation promoted a reduction in overall systolic blood pressure (SBP), awake and morning SBP, as well as a reduction in morning surge in patients with cardiovascular autonomic neuropathy and type 1 diabetes mellitus. Additionally, we observed associations between 25(OH)D levels, CAN, and nocturnal dipping in patients with type 1 diabetes mellitus. The improvement in CAN parameters after VD supplementation was mainly observed in the non-dipper group, which had a higher prevalence of CAN at the beginning of the study, minimizing the existing differences between this group and the dipper group found at baseline. Finally, in spite of need to be confirmed by large, randomized trials, our findings suggests that VD in higher doses could be a potential approach in the treatment of type 1 diabetes mellitus patients with CAN.

## Data Availability

The datasets generated during and/or analyzed during the current study are available from the corresponding author on reasonable request.
